# Development of a CDK10/CycM *in vitro* Kinase Screening Assay and Identification of First Small-Molecule Inhibitors

**DOI:** 10.3389/fchem.2020.00147

**Published:** 2020-02-27

**Authors:** Thomas Robert, Jared L. Johnson, Roxane Guichaoua, Tomer M. Yaron, Stéphane Bach, Lewis C. Cantley, Pierre Colas

**Affiliations:** ^1^Laboratory of Integrative Biology of Marine Models, Station Biologique de Roscoff, Sorbonne Université/CNRS, Roscoff, France; ^2^Kinase Inhibitor Specialized Screening Facility (KISSf), Station Biologique de Roscoff, Sorbonne Université/CNRS, Roscoff, France; ^3^Meyer Cancer Center, Weill Cornell Medicine, New York, NY, United States

**Keywords:** CDK10, Cyclin M, CDK10tide, screening assay, kinase inhibitors, NVP-2

## Abstract

Cyclin-dependent kinases (CDKs) constitute a family of 20 serine/threonine protein kinases that play pivotal roles in the regulation of numerous important molecular and cellular processes. CDKs have long been considered promising therapeutic targets in a variety of pathologies, and the recent therapeutic success of CDK4/6 inhibitors in breast cancers has renewed interest in their therapeutic potential. Small-molecule inhibitors have been identified for every human CDK, except for CDK10. The only recent discovery of an activating cyclin (CycM) for CDK10 enabled us to identify its first phosphorylation substrates and gain insights into its biological functions. Yet, our knowledge of this kinase remains incomplete, despite it being the only member of its family that causes severe human developmental syndromes, when mutated either on the cyclin or the CDK moiety. CDK10 small-molecule inhibitors would be useful in exploring the functions of this kinase and gauging its potential as a therapeutic target for some cancers. Here, we report the identification of an optimized peptide phosphorylation substrate of CDK10/CycM and the development of the first homogeneous, miniaturized CDK10/CycM *in vitro* kinase assay. We reveal the ability of known CDK inhibitors, among which clinically tested SNS-032, riviciclib, flavopiridol, dinaciclib, AZD4573 and AT7519, to potently inhibit CDK10/CycM. We also show that NVP-2, a strong, remarkably selective CDK9 inhibitor is an equally potent CDK10/CycM inhibitor. Finally, we validate this kinase assay for applications in high-throughput screening campaigns to discover new, original CDK10 inhibitors.

## Introduction

Cyclin-dependent kinases (CDKs) form a family of 20 serine/threonine protein kinases that are involved in the regulation of multiple biological processes, such as cell division, apoptosis, transcription, mRNA splicing, metabolism, ciliogenesis, etc. (Lim and Kaldis, 2013). CDKs have long been considered promising therapeutic targets for a variety of pathologies, notably cancers. Hence, massive efforts have been dedicated to the discovery and development of CDK small-molecule inhibitors as drug candidates (Roskoski, 2019). However, most inhibitors that have been developed so far inhibit multiple CDKs (not mentioning other protein kinases) and they have produced disappointing outcomes in clinical trials, partly due to their lack of specificity (Asghar et al., 2015). The recent tremendous therapeutic success of highly selective CDK4/6 inhibitors against hormone-dependent metastatic breast cancers and their promising activity against other solid tumors (Schettini et al., 2018) have demonstrated that CDKs can be valuable therapeutic targets, provided that they are addressed by selective molecules.

Small-molecule inhibitors have been identified against all CDKs except CDK10, either through direct screening campaigns, or through profiling assays that determine the ability of a given CDK inhibitor to target other CDKs and other kinases. The “Illuminating the Druggable Genome” (IDG) program, aimed at identifying therapeutic opportunities in the human genome, classifies human proteins according to the level of knowledge available on each of them (Oprea et al., 2018). Strikingly, among the CDK family, only two CDKs currently appear in the class of proteins for which no small-molecule inhibitor is available: CDK20 and CDK10^1^. However, a sub-nanomolar inhibitor that targets a few CDKs including CDK20 has recently been reported (Mueller et al., 2016).

Although it was discovered in the pre-genomic era, CDK10's biological functions have long remained mysterious. The identification of cyclin M as a CDK10 binding and activating partner enabled us to show that this kinase phosphorylates the ETS2 oncoprotein and controls its stability (Guen et al., 2013), and that it regulates actin network architecture and ciliogenesis (Guen et al., 2016), (reviewed in Guen et al., 2017). Yet, we still know little about CDK10/CycM, although it stands out as the only member of its family responsible for severe human developmental syndromes, when mutated either on the cyclin (Unger et al., 2008) or the CDK moiety (Windpassinger et al., 2017; Guen et al., 2018). CDK10 small-molecule inhibitors would complement the classical reverse genetics toolbox in exploring biological functions of this protein kinase. They may also confirm the therapeutic interest of CDK10/CycM as a target in colorectal adenocarcinomas, as recently suggested by a reverse genetics approach (Weiswald et al., 2017). However, to date, no screening assays or inhibitors for CDK10 have been reported.

Here, we describe the identification of an optimized peptide phosphorylation substrate of CDK10/CycM and the development of a first homogeneous, miniaturized *in vitro* kinase assay. We also unveil the ability of known CDK inhibitors, some of which tested in clinical trials, to potently inhibit CDK10/CycM *in vitro*. Finally, we show that this assay is amenable to high-throughput screening campaigns.

## Materials and Methods

### Insect Cell Expression Plasmids

We constructed a plasmid enabling the production of a bacculovirus that co-expresses GST-CDK10 and Strep2-CycM recombinant proteins. We digested pVL1393:Strep2-CycM (Guen et al., 2013) with BamHI and NotI and ligated the insert into BamHI/NotI-cut pFastBacDual (ThermoFisher Scientific). Then, we amplified the GST-CDK10 coding sequence from pGEX6P1:CDK10 (Guen et al., 2013) using the oligonucleotides 5′-ATATCTCGAGACCATGTCCCCTATACTAGGTTATTG-3′ and 5′-ATATATGCATTCAGGGTTTACAGCGCTTGC-3′ that contain a XhoI and a NsiI site, respectively. We ligated the PCR product into XhoI/NsiI-cut pFastBacDual:Strep2-CycM. To produce a kinase dead version of CDK10 (D181N), we performed a site-directed mutagenesis using a QuickChange XL kit (Agilent) and the oligonucleotides 5′-AAGACAGCGAATTTCGGCCTGGCCCGGGCC-3′ and 5′-GCCGAAATTCGCTGTCTTCACACAACCC-3′.

### Protein Expression and Purification

We produced GST-CDK10(wt or kd)/Strep2-CycM in Sf9 cells using the Bac-to-Bac baculovirus expression system (ThermoFisher Scientific) according to the manufacturer's instructions. We resuspended the cells in a buffer containing 1x PBS, 1 mM EDTA, 1x CLAPA protease inhibitor cocktail (2 μg/ml each of chymostatin, leupeptin, antipain and pepstatin), 1 mM PMSF. We sonicated the cell suspension (Branson sonifier 150) and spun the lysate 30 min at 15,000g. We filtered the lysate through a 0.45 μm cellulose acetate membrane. We loaded the filtrate onto a glutathione sepharose 4B column (GSTrap 4B), using an ÄktaPrime FPLC device (GE Healthcare). After washing with 1x PBS, 1 mM EDTA, 1 mM DTT, we eluted the heterodimers using 10 mM of reduced glutathione. We concentrated the eluates using an Amicon Ultra-15 centrifugal filter and we added glycerol (20% final concentration) to store the samples at −80°C. We determined the total protein concentration using a Bradford assay (Bio-Rad). We used baculoviruses directing the expression of MBP-CDK9 (1-372) and GST-CycT1 (1-298) (Baumli et al., 2008) and we produced Sf9 lysates using a very similar procedure as described above. We directly loaded the lysates onto glutathione agarose beads (Sigma Aldrich) and incubated for 1h under agitation. We washed the beads with 1x PBS, 1 mM DTT, 1x CLAPA cocktail, 1 mM PMSF and we eluted the heterodimer using 20 mM reduced glutathione in 1x PBS, 1 mM DTT. We added glycerol (15% final concentration) and stored the samples at −80°C.

### Positional Scanning Peptide Library Assay

To determine the substrate motif of CDK10, we performed *in vitro* kinase assays with recombinant GST-CDK10/Strep2-CycM on the peptide substrate library in the presence of ATP[γ-^32^P]. We carried out these reactions in 10 mM MgCl_2_, 25mM Tris-HCl pH 7.5, 1 mM EGTA, 1 mM DTT, and 50 μg/mL heparin at 30°C for 90 min. The peptides, which are biotinylated at their C-termini, were blotted onto streptavidin-conjugated membranes and imaged with a Typhoon FLA 7000 phosphorimager. Detailed information on the protocol is provided elsewhere (Turk et al., 2006). We quantified the spot densities from the blot array and we normalized by each row. We used these values to score the amino acid sequence surrounding each identified phospho-site and we applied them to predict highest scoring *in vitro* substrate peptides.

### Protein Kinase Assays

#### CDK10/CycM

We performed the kinase reaction assays in white opaque, flat-bottom 384-well microplates (Optiplate, Perkin Elmer) in a total volume of 6 μL, adding kinase reaction buffer (final concentrations: 25 mM Tris-HCl pH7.5, 10 mM MgCl_2_, 1 mM EGTA, 1mM DTT, 50 μg/mL Heparin, 3 μg/mL BSA), DMSO 1% (or molecules diluted in 1% DMSO), recombinant purified GST-CDK10/Strep2-CycM (50 nM) and peptide substrate (150 μM) (except when indicated otherwise in figure legends), and ATP 10 μM (except for the K_m, ATP_ determination assay). We incubated the plates 30 min at 30°C and we measured the protein kinase activity using the ADP-Glo kinase assay (Promega). We added 6 μL of ADP-Glo reagent and we incubated the plates 50 min at room temperature. We then added 12 μL of kinase detection reagent and we incubated the plates 60–90 min at room temperature. We mildly agitated the plates during all incubation steps. We measured the luminescence using an Envision plate reader (Perkin Elmer). All measurements were performed in triplicates except for the measurements of the IC_50_ values, which were performed in duplicates. For the validation of the screening assay in a 384-well plate, we used columns 1 and 24 for a no-substrate control and we filled columns 2–23 in an interleaved format of high (DMSO), low (NVP-2) and no (empty wells) signals, leaving the first and last two rows empty. We filled the plate using a Janus Expanded automated liquid handling system (Perkin Elmer).

#### CDK9/CycT1

We followed a similar procedure, using 80 μM of CDK7/9tide peptide (YSPTSPSYSPTSPSYSPTSPSKKKK) as a substrate and 17 nM of enzyme.

## Results

### Identification of Peptide Phosphorylation Substrates

**Figure 1:**
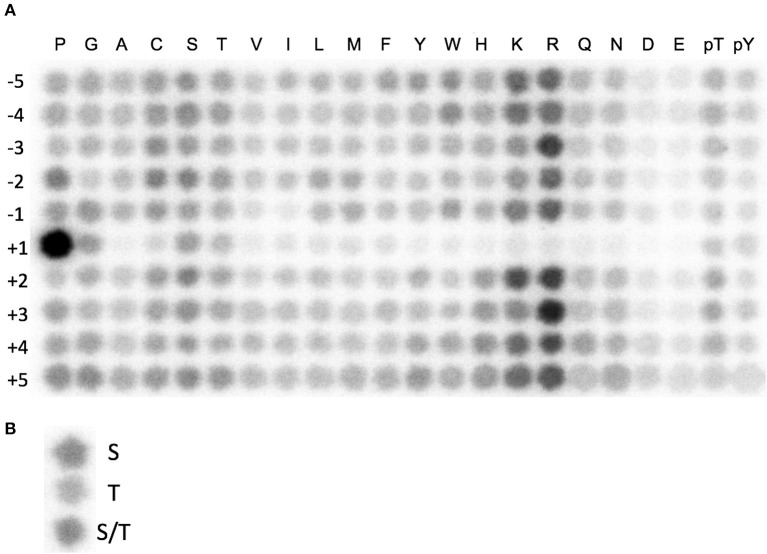
Identification of a CDK10/CycM optimal phosphorylation motif. **(A)** Combinatorial peptide blot array submitted to a CDK10/CycM *in vitro* kinase reaction in presence of ATP[γ-^32^P]. Amino acid coordinates (−5 to +5) refer to the positions relative to a Serine, Threonine, or a mixture of both phospho-acceptor amino acids. **(B)** Determination of the preferred phospho-acceptor amino acid. Random sequence peptides that contain a central serine, threonine, or mixture of both were spotted and treated as in **(A)**.

We set out to develop a non-radioactive *in vitro* CDK10/CycM kinase assay amenable to high-throughput screening campaigns. Based on prior successful experiences with other kinases, we opted for a luminescent assay that quantifies ADP produced by a kinase reaction with a phosphorylation substrate (Zegzouti et al., 2009). Using recombinant purified GST-CDK10/Strep2-CycM produced in insect cells, we first tested its activity on recombinant purified ETS2 and PKN2 proteins, two phosphorylation substrates that we had previously discovered, and then ETS2- and PKN2- derived peptides containing the residues phosphorylated by CDK10/CycM (Guen et al., 2013, 2016). We failed to detect kinase activity in all cases (*not shown*). We then utilized a combinatorial peptide library method, involving an array of 180 peptides where the 20 natural amino acids are scanned across nine positions neighboring the serine/threonine phospho-acceptor. We performed *in vitro* radioactivity-based kinase assays to determine the optimal amino acid sequence for phosphorylation by CDK10/CycM, referred to as its phosphorylation motif (Hutti et al., 2004). We identified XXRXXSP(KR)RXX as the optimal phosphorylation motif (Figure 1A). This indicates that CDK10 is a proline-directed basophilic kinase that favors arginine at −3 and +3. We also examined the “zero” position to see if CDK10 distinguishes between serine and threonine as the phospho-acceptor and it showed slightly more preference for serine than threonine (Figure 1B). This data guided the design of two substrate peptide candidates for CDK10: Jar 2 (KKRRRSPKRKR) that directly resulted from the raw spot intensities, and Jar1 (GNRPGSPKRGG) obtained after signal normalization.

**Figure 2:**
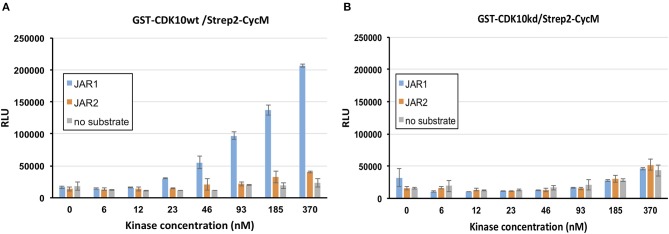
Identification of a peptide phosphorylation substrate of CDK10/CycM in a luminescent kinase assay. *in vitro* kinase activity of GST-CDK10*wt*/Strep2-CycM **(A)** or GST-CDK10*kd*/Strep2-CycM **(B)** against Jar1 and Jar2 peptides, using increasing concentrations of kinase (0–370 nM) and 150 μM of peptides. Kinase assays were performed in triplicates. RLU, relative light unit.

We tested Jar1 and Jar2 synthetic peptides as substrate candidates in the luminescent kinase assay, using either GST-CDK10*wt*/Strep2-CycM or GST-CDK10*kd*/Strep2-CycM (the kinase dead mutant bearing a D181N substitution that abolishes ATP binding) as a control (van den Heuvel and Harlow, 1993). As shown in Figure 2A, the phosphorylation of Jar1 by GST-CDK10wt/Strep2-CycM was readily detectable and increased with increasing concentrations of the kinase. In contrast, no significant signal was detected using GST-CDK10kd/Strep2-CycM (Figure 2B), thus ruling out that the phosphorylation detected with the wild-type kinase is due to a contaminating, co-purified protein kinase. We observed no significant phosphorylation of the Jar2 peptide, and we thus retained Jar1 (thereafter renamed CDK10tide) as a substrate for further studies.

### Optimization of the Kinase Assay and Determination of the K_m, ATP_

**Figure 3:**
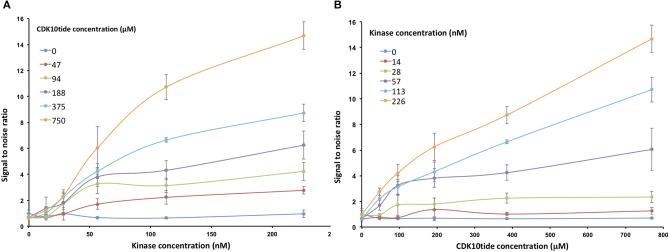
Optimization of the luminescent CDK10/CycM kinase assay. *in vitro* kinase activity of increasing concentrations of GST-CDK10*wt*/Strep2-CycM (0–226 nM) against increasing concentrations of CDK10tide (0–750 μM). Signal to noise ratios were calculated by dividing each luminescence value with that obtained without peptide substrate. **(A)** Signal to noise ratios obtained with increasing concentrations of kinase are plotted for each tested concentration of peptide; **(B)** signal to noise ratios obtained with increasing concentrations of peptide are plotted for each tested concentration of kinase. Kinase assays were performed in triplicates.

**Figure 4:**
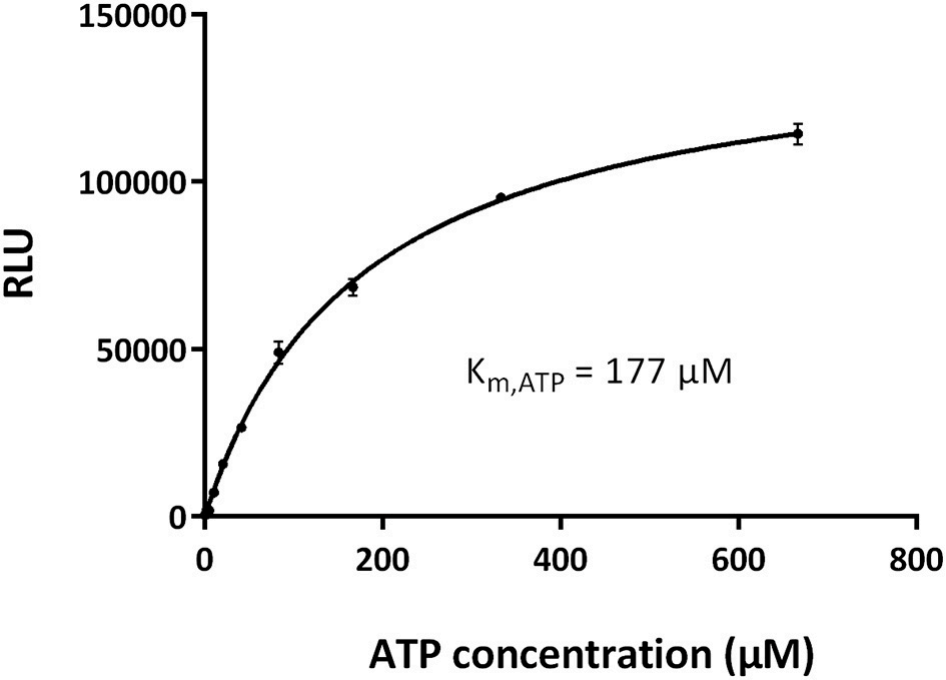
Determination of the K_m, ATP_ of CDK10/CycM. Michaelis-Menten plot of GST-CDK10/Strep2-CycM kinase activity measured with increasing concentrations of ATP (0–667 μM). Kinase assays were performed in triplicates. The K_m, ATP_ value, which represents the ATP concentration required to reach the half-maximal kinase activity, was obtained using the GraphPad Prism software.

To optimize the signal-to-noise ratio, we performed the kinase assay by varying the concentrations of GST-CDK10wt/Strep2-CycM and CDK10tide. As shown in Figure 3A, for each concentration of CDK10tide tested, the signal increased almost linearly with the kinase concentration, except for the lowest concentrations of CDK10tide for which a plateau was observed at the highest kinase concentrations. Likewise, for each concentration of kinase tested, the signal increased with CDK10tide concentration, except for the lowest kinase concentrations (Figure 3B). We set the optimal concentrations of kinase and CDK10tide at 50 nM and 150 μM, respectively, which offers a good compromise between an acceptable signal-to-noise ratio and a limited consumption of recombinant kinase. Using these concentrations, we then determined the K_m, ATP_ of GST-CDK10/Strep2-CycM. To this end, we performed the kinase assay using different concentrations of ATP (Figure 4). We measured a K_m, ATP_ of 177 μM, a rather high but not uncommon value for CDKs (Knight and Shokat, 2005).

### Testing of CDK Inhibitors

Most CDK inhibitors target multiple CDKs. We thus tested a first panel of common pan-CDK inhibitors (olomoucine, roscovitine, CR8, meriolin, flavopiridol) at high concentrations (1 and 10 μM) against the *in vitro* kinase activity of GST-CDK10/Strep2-CycM. We also tested palbociclib, one of the CDK4/6-inhibiting therapeutic molecules. None of these compounds but flavopiridol produced an inhibition greater than 50% at 1 μM (data not shown).

**Figure 5:**
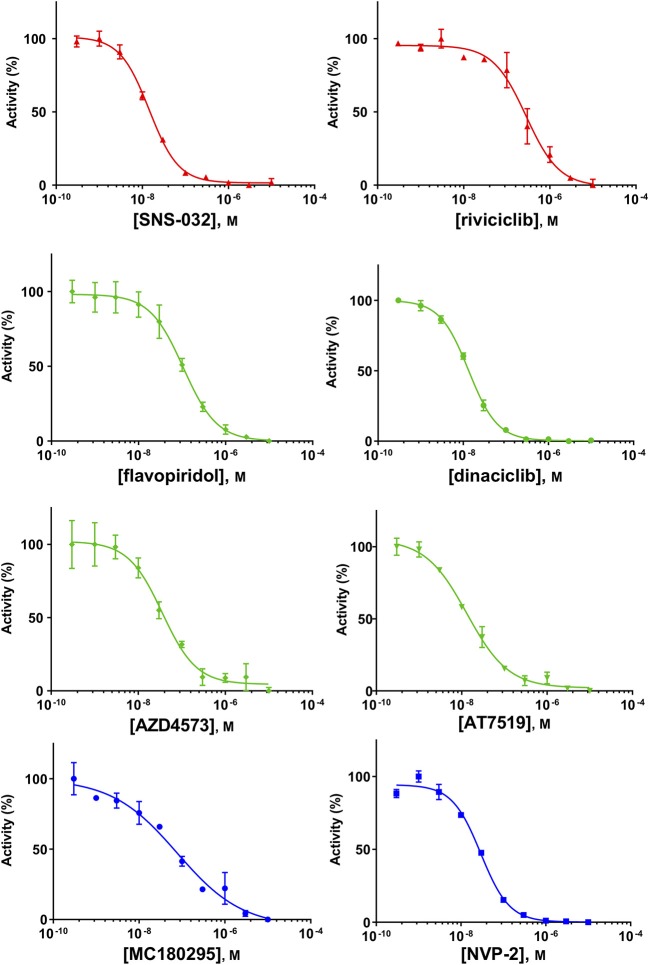
Determination of the IC_50_ values of a panel of CDK9 inhibitors against CDK10/CycM. A panel of CDK9 inhibitors was tested at different concentrations against GST-CDK10/Strep2-CycM. Results are expressed as percentages of maximal kinase activity, measured in absence of inhibitor. Mean percentages are reported ± SD. Kinase assays were performed in duplicates. IC_50_ values were determined using the GraphPad Prism software. Red: inhibitors for which clinical trials have been completed or terminated; green: inhibitors that are being (or will be soon) tested in clinical trials; blue: recently discovered inhibitors.

Because of the high inhibitory potency of flavopiridol against CDK9 (Chao et al., 2000), and considering that CDK10 is phylogenetically closer to CDK9 and other so-called “transcriptional CDKs” than to “cell-cycle CDKs” (Malumbres et al., 2009), we decided to test a panel of known potent CDK9 inhibitors against the *in vitro* kinase activity of GST-CDK10/Strep2-CycM in dose-response assays, to determine half-maximal inhibitory concentrations (IC_50_'s). We first examined flavopiridol (Blagosklonny, 2004), dinaciclib (Parry et al., 2010), SNS-032 (Chen et al., 2009), AZD4573 (Cidado et al., 2019), AT7519 (Squires et al., 2009) and riviciclib (Joshi et al., 2007), six inhibitors that have been or are still tested in various clinical trials. As shown in Figure 5, Figure S1, Table 1, all six molecules potently inhibited GST-CDK10/Strep2-CycM, as much as they inhibited MBP-CDK9/GST-CycT1 (dinaciclib, SNS-032), ca. 5x less (flavopiridol, AZD4573, AT7519), or 40x less (riviciclib). We also tested two new highly selective CDK9 inhibitors dubbed MC180295 (Zhang et al., 2018) and NVP-2 (Olson et al., 2018). The former inhibited GST-CDK10/Strep2-CycM ca. 10x less than MBP-CDK9/GST-CycT1, and the latter almost as much.

**Table 1:** IC_50_ values of a CDK inhibitor panel against CDK9 and CDK10.

		**CDK9/CycT1** **(published)**	**CDK9/CycT1** **(measured herein)**	**CDK10/CycM** **(measured herein)**
SNS-032	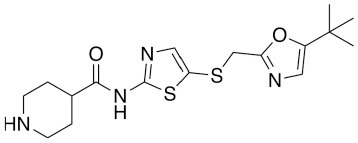	4 nM^a^	6 nM	14 nM
riviciclib	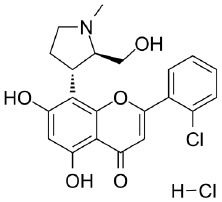	20 nM^b^	7 nM	280 nM
flavopiridol	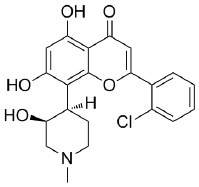	6 nM^c^	15 nM	107 nM
dinaciclib	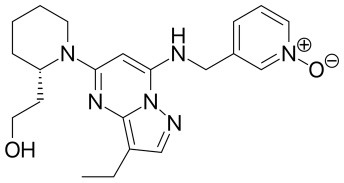	4 nM^d^	13 nM	13 nM
AZD4573	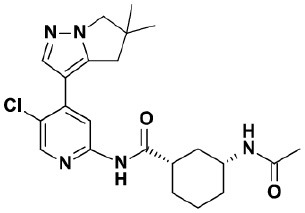	3 nM^e^	5 nM	36 nM
AT7519	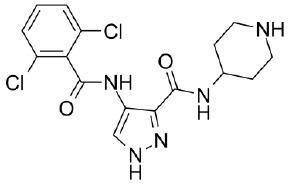	<10 nM^f^	3 nM	13 nM
MC180295	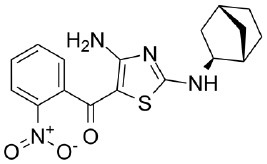	5 nM^g^	6 nM	77 nM
NVP-2	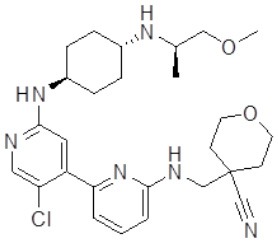	0.5 nM^h^	6 nM	29 nM

*Red: inhibitors for which clinical trials have been completed or terminated; green: inhibitors that are being (or will be soon) tested in clinical trials; blue: recently discovered inhibitors*.

a Conroy et al. (2009);

b Joshi et al. (2007);

c Chao et al. (2000);

d Parry et al. (2010);

e Cidado et al. (2019);

f Squires et al. (2009);

g Zhang et al. (2018);

h *Olson et al. (2018)*.

### Validation of the CDK10/CycM Kinase Assay for High-Throughput Screening Applications

**Figure 6:**
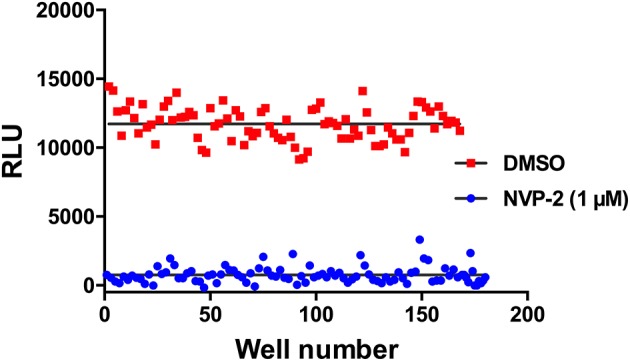
Signal variability assessment of the CDK10/CycM kinase activity in a high-throughput screening setting. Columns of a 384-well plate were filled in an interleaved fashion as to alternate low (1 μM NVP-2) and high (DMSO) luminescence signals. Mean background signal obtained without substrate was subtracted from each value.

Having developed this luminescent CDK10/CycM kinase activity assay in the 384-well plate format, we set out to determine whether it is amenable to high-throughput screening campaigns that will be carried out to discover new, original CDK10 inhibitors. To this end, we performed a variability assessment on the maximum signal (obtained in presence of the DMSO solvent) and the minimum signal (obtained in presence of 1 μM NVP-2, which produces a close to total inhibition of the kinase; Figure 5). As shown in Figure 6, the assay showed a good level of robustness in a screening setting, with a Z-factor of 0.51.

## Discussion

The discovery of CDK10/CycM small-molecule inhibitors has been hindered so far by the absence of a strong peptide substrate for *in vitro* assays. Our work provides yet another demonstration of the power of the Positional Scanning Peptide Library (PSPL) assay, which has enabled the design of a peptide substrate of CDK10/CycM whose phosphorylation is readily detected by a luminescent assay. The PSPL method has been successfully applied to many kinases and the full potential of this technique has not been totally utilized, considering that small-molecule inhibitors remain to be discovered for more than 200 kinases, according to the IDG target development level survey (Oprea et al., 2018).

None of the pan-CDK inhibitors tested here were shown to be significantly active against CDK10/CycM except for flavopiridol, one of the most investigated CDK inhibitor, which has been tested in more than 60 clinical trials so far. The fact that flavopiridol potently inhibits CDK9 and that CDK10 is more closely related to “transcriptional CDKs” than to “cell cycle CDKs” prompted us to examine the ability of other CDK9 inhibitors to inhibit CDK10/CycM. All eight CDK9 inhibitors tested (flavopiridol, dinaciclib, SNS-032, AZD4573, AT7519, riviciclib, MC180295 and NVP-2) proved to be strong CDK10 inhibitors. Six of these inhibitors have been (Asghar et al., 2015), are currently, and/or will soon be evaluated in clinical trials (Table 2).

**Table 2:** Programmed or ongoing clinical trials of flavopiridol, dinaciclib, AZD4573 and AT7519 (as of January 2020).

**Inhibitor**	**Clinical phase**	**Medical condition**	**Combination treatment**	**Status**
flavopiridol *(aka alvocidib)*	1	Relapsed/refractory AML	Venetoclax	Recruiting
	1b/2	Myelodysplastic syndromes	Decitabine	Recruiting
	1	Newly diagnosed AML	Cytarabine/Daunorubicin	Recruiting
	1	AML	Cytarabine/Mitoxantrone/Daunorubicin	Recruiting
	2	Biomarker-driven AML study	Cytarabine/Mitoxantrone	Recruiting
	1	Advanced solid tumors	–	Recruiting
dinaciclib	1	Relapsed/refractory AML	Venetoclax	Recruiting
	1	Hematologic malignancies	Pembrolizumab	Active
	1	Advanced solid tumors	Veliparib	Recruiting
	2	Stage IV melanomas	–	Active
	1b	Advanced breast cancer	Pembrolizumab	Recruiting
AZD4573	1	Hematologic malignancies	–	Recruiting
AT7519	1	Metastatic solid tumors	Onalespib	Active

*AML, acute myeloid leukemia; information. Source: www.clinicaltrials.gov*.

Although a recent study argued that CDK10 is a candidate therapeutic target for colorectal adenocarcinomas (Weiswald et al., 2017), a number of reports indicate that CDK10 acts as a tumor suppressor in various cancers of the digestive system, including biliary tract cancer (Yu et al., 2012), hepatocellular carcinomas (Zhong et al., 2012), gastric carcinomas (Zhao et al., 2017; You et al., 2018), and in nasopharyngeal carcinomas (You et al., 2013), gliomas (Li et al., 2018) and advanced breast cancers (You et al., 2015). These contradictory findings could be explained by the fact that ETS2, whose stability is controlled in part by CDK10/CycM phosphorylation (Guen et al., 2013), can function either as an oncogene or a tumor suppressor depending on various factors such as cellular context and p53 status (Martinez, 2016). Moreover, CDK10 is subject to a complex splicing process (Crawford et al., 1999), and different splice variants might exert different and possibly opposite roles in cancer, as observed with other important proteins in tumorigenesis (Christofk et al., 2008; Kaida et al., 2012).

Our findings that clinically tested molecules potently inhibit CDK10/CycM *in vitro* might help explain previous failures in clinical trials and will be useful information to clinical investigators in charge of ongoing or imminent trials. The main rationale of testing pan-CDK inhibitors in cancer patients lies in the ability of these molecules to inhibit both cell-cycle-driving CDKs (such as CDK1 and 2) and transcription-driving CDKs (such as CDK7 and 9), which, in the latter case, causes the loss of short-lived mRNAs coding for anti-apoptotic proteins (Blagosklonny, 2004). A future challenge for drug development will be to design small molecules that target cell-cycle CDKs and transcriptional CDKs without inhibiting CDK10 and other CDKs that can act as tumor suppressors, such as CDK12 (Paculova and Kohoutek, 2017).

Similarly, a future challenge for chemical biology purposes will be to design CDK10 inhibitors that do not inhibit (or inhibit much less) CDK9 and other CDKs. The selectivity of NVP-2 was recently characterized in detail, using a competition binding assay with a panel of 468 recombinant purified kinases (that did not include CDK10) followed by *in vitro* enzymatic assays on the kinase hits, and a target engagement assay on cell lysates (Olson et al., 2018). The former approach demonstrated an exquisite selectivity toward CDK9, and the latter revealed an exclusive, strong engagement of CDK9 and CDK10. So far, such a level of selectivity has only been achieved with CDK4/CDK6 inhibitors (palbociclib, alvociclib, ribociclib), the first approved CDK inhibitors that are meeting a great success in metastatic breast cancer treatment (Schettini et al., 2018). Hence, NVP-2 represents a highly promising starting point to design analogs that will hopefully inhibit selectively CDK10/CycM. In complement to this rational approach, the validation of our *in vitro* CDK10/CycM kinase assay for high-throughput applications has enabled us to launch screening campaigns of large chemical collections, which are delivering other promising small-molecule inhibitors. If selective enough, such molecules will considerably help the exploration of the functions of CDK10/CycM, and they will also allow to determine whether it represents an interesting therapeutic target for some cancers.

## Data Availability Statement

All datasets generated for this study are included in the article/Supplementary Material.

## Author Contributions

JJ and TY identified the combinatorial peptide substrates and designed optimized peptides, with supervision from LC. TR and RG performed all other experiments. SB supervised the screening platform. PC performed the mutagenesis, supervised the whole project, and wrote the manuscript. All authors contributed to manuscript revision, read, and approved the submitted version.

### Conflict of Interest

LC is a founder and member of the SAB of Agios Pharmaceuticals and of Petra Pharmaceuticals. These companies are developing novel therapies for cancer. LC's laboratory also receives some financial support from Petra Pharmaceuticals. SB is a founder and a member of the SAB of SeaBeLife Biotech, which is developing novel therapies for liver and kidney acute disorders. The remaining authors declare that the research was conducted in the absence of any commercial or financial relationships that could be construed as a potential conflict of interest.

## Footnotes

^1^https://druggablegenome.net

## Supplementary Material

Figure S1 Determination of the IC_50_ values of a panel of CDK9 inhibitors against CDK9/CycT1. A panel of CDK9 inhibitors was tested at different concentrations against MBP-CDK9/ GST-CycT1. Results are expressed as percentages of maximal kinase activity, measured in absence of inhibitor. Mean percentages are reported ±SD. Kinase assays were performed in duplicates. IC_50_ values were determined using the GraphPad Prism software.
