# Supplementary figures and images for: Development of a CDK10/CycM in vitro Kinase Screening Assay and Identification of First Small-Molecule Inhibitors
Source: Front Chem. 2020 Feb 27;8:147. doi: 10.3389/fchem.2020.00147 (PMC7056863; doi:10.3389/fchem.2020.00147)

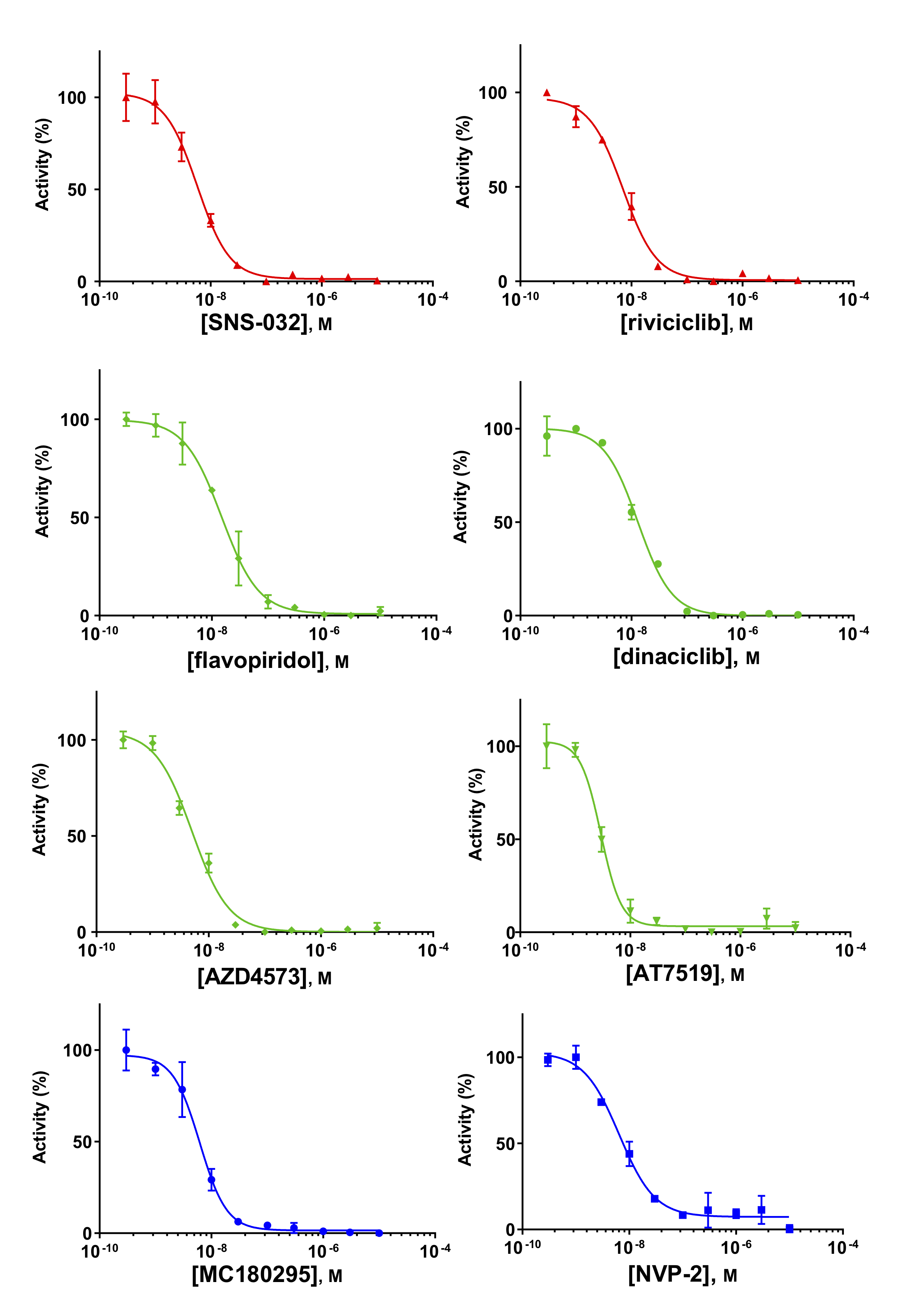

Supplement: Figure S1 — Determination of the IC50 values of a panel of CDK9 inhibitors against CDK9/CycT1. A panel of CDK9 inhibitors was tested at different concentrations against MBP-CDK9/ GST-CycT1. Results are expressed as percentages of maximal kinase activity, measured in absence of inhibitor. Mean percentages are reported ±SD. Kinase assays were performed in duplicates. IC50 values were determined using the GraphPad Prism software. [file Image_1.TIFF]
